# Supplementary material for: In Vitro Evaluation of Antiprotozoal Properties, Cytotoxicity Effect and Anticancer Activity of New Essential-Oil Based Phytoncide Mixtures
Source: Molecules. 2023 Feb 1;28(3):1395. doi: 10.3390/molecules28031395 (PMC9921295; doi:10.3390/molecules28031395)
Supplement: Supplementary file 1 [file molecules-28-01395-s001.zip › molecules-2169604-supplementary.pdf]

Supplementary Materials

In Vitro Evaluation of Antiprotozoal Properties, Cytotoxicity Effect and Anticancer Activity of New Essential-Oil Based Phytoncide Mixtures

Table of content

Figure S1. MS Spectrum of unknown compound (nr 8) in clove EO: 1

Figure S2. Evaluation of cytotoxicity of test oils (1 mg/mL) to normal human fibroblasts after 48 h incubation, 2

All presented in the publication GC-MS chromatograms are available under following link:

https://drive.google.com/file/d/1LfmYecuE3QSYoeVn43-ccu0O2s_UcS-L/view?usp=share_link

The NMR spectra presented in publication are available under following link:

https://drive.google.com/file/d/1zkAnN77n6CRTGrYKynBj8GmKxt3H45I9/view?usp=share_link

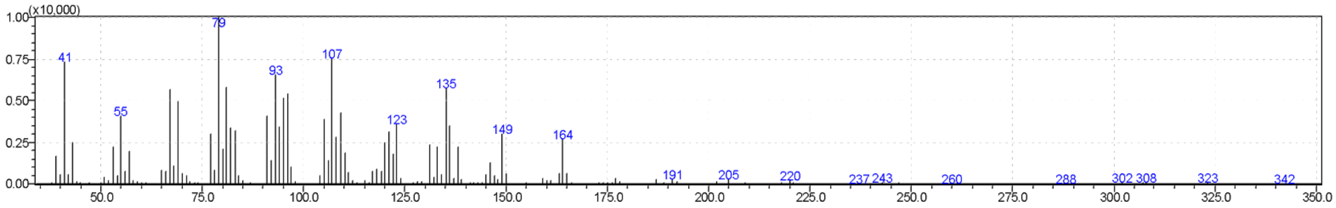

Figure S1. MS Spectrum of unknown compound (nr 8) in clove EO:.

CONTROL

CONTROL +H_2O_2 (2mM)

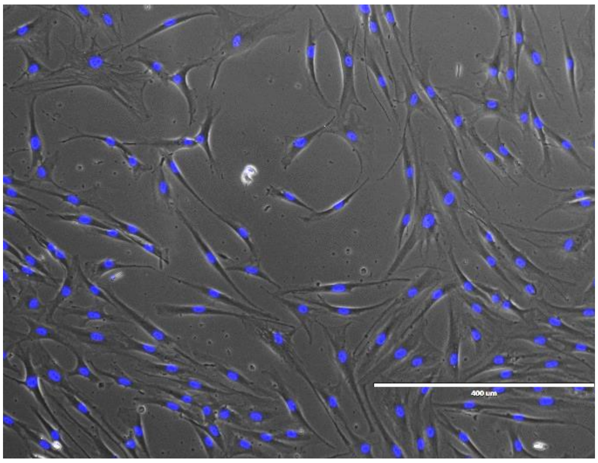

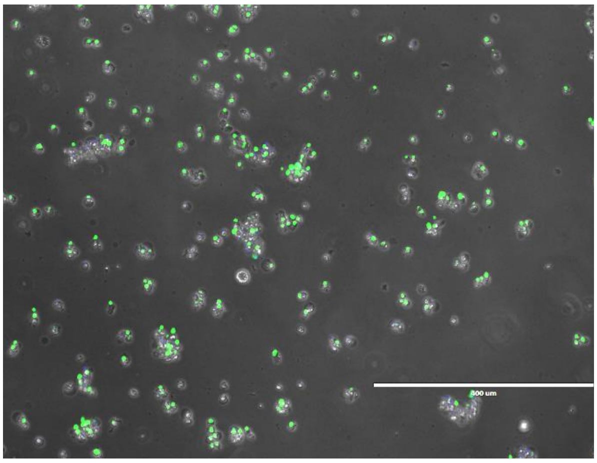

essential oil of Ceylon cinnamon

essential oil garlic essential oil

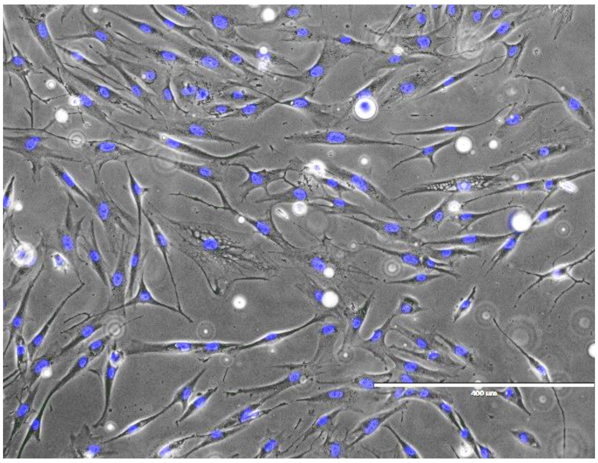

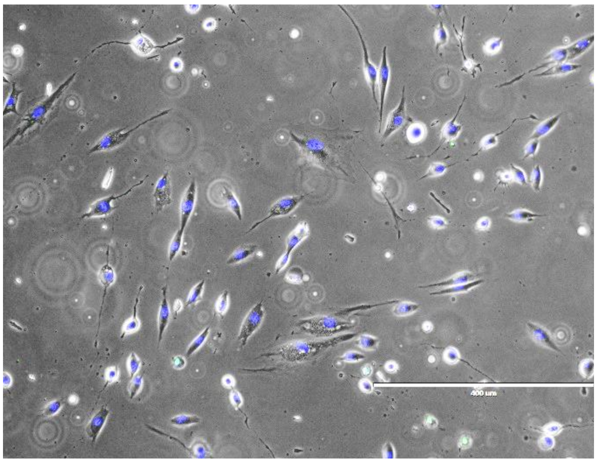

clove essential oil

rosemary essential oil

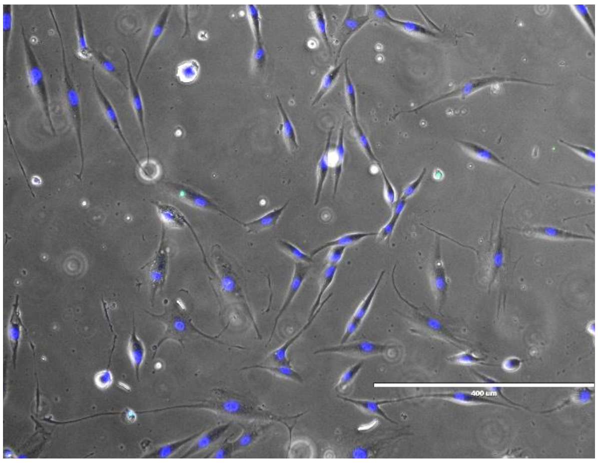

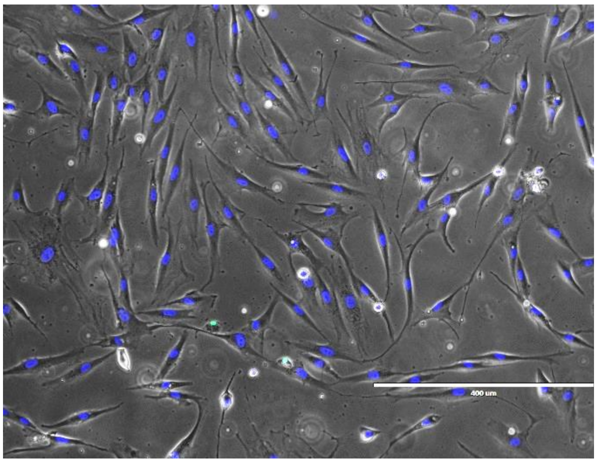

Figure S2. Evaluation of cytotoxicity of test oils (1 mg/mL) to normal human fibroblasts after 48 h incubation, live-dead staining, staining kit: ReadyProbes™ Cell Viability Imaging Kit, Blue/Green, blue - live, green - dead cells. Evos FL microscope, objective magnification 10x, fluorescence: DAPI: Ex: 357/44 Em: 447/60; GFP: Ex: 482/25 Em: 524/24.
